# MicroRNA-140-5p inhibits salivary adenoid cystic carcinoma progression and metastasis via targeting survivin

**DOI:** 10.1186/s12935-019-1018-4

**Published:** 2019-11-16

**Authors:** Zhu Qiao, Yue Zou, Hu Zhao

**Affiliations:** 1 Stomatology Second Unit, Baoding No.1 Central Hospital, Baoding, 071000 Hebei China; 2 Central Sterile Supply Department, Baoding No.1 Central Hospital, Baoding, Hebei China

**Keywords:** SACC, Proliferation, Apoptosis, Invasion, miR-140-5p, Survivin

## Abstract

**Background:**

Salivary adenoid cystic carcinoma (SACC) is one of the most frequent carcinomas derived from the salivary gland. Growing evidence implied the involvement of microRNAs (miRNAs) in SACC progression and metastasis. This study aimed to determine the regulatory role of miR-140-5p in SACC progression and metastasis and to explore the underlying mechanisms.

**Materials and methods:**

MiR-140-5p and survivin mRNA expression levels were determined by quantitative real-time PCR; protein levels were evaluated by western blot assay; cell proliferation, growth, invasion, apoptosis and caspase-3 activity were evaluated by respective in vitro functional assays; xenograft nude mice model was used to assess the in vivo tumor growth; a luciferase reporter assay determined the interaction between miR-140-5p and survivin.

**Results:**

MiR-140-5p overexpression suppressed SACC cell proliferation and invasion, induced cell apoptosis and inhibited in vivo tumor growth of SACC cells. The loss-of-function studies showed that miR-140-5p knockdown enhanced SACC cell proliferation and invasion, inhibited cell apoptosis and led to an accelerated in vivo tumor growth. The bioinformatics prediction and luciferase reporter assay revealed that miR-140-5p directly targeted survivin 3′ untranslated region, and survivin was inversely regulated by miR-140-5p. Knockdown of survivin exerted tumor-suppressive effects on SACC cells, while enforced expression of survivin counteracted the tumor-suppressive actions of miR-140-5p overexpression in SACC cells. Mechanistically, miR-140-5p modulated the protein expression levels of apoptosis- and epithelial-mesenchymal transition-related mediators as well as matrix metallopeptidase-2/-9 via targeting survivin. More importantly, the down-regulation of miR-140-5p and the up-regulation of survivin were detected in the SACC clinical tissues, and miR-140-5 expression was inversely correlated with survivin mRNA expression level in SACC tissues.

**Conclusion:**

Our data indicated that miR-140-5p suppressed SACC cell proliferation and invasion, induced cell apoptosis via regulating survivin expression. The present study provide evidence that that miR-140-5p could be a promising target for treating SACC, which requires further investigations.

## Background

Salivary adenoid cystic carcinoma (SACC) is one of the most frequent carcinomas derived from the salivary gland [1]. The main treatments for SACC include surgical resection in combination with radiotherapy and/or chemotherapy [2, 3]. However, more than 30% of the SACC patients after primary treatments had local or distant recurrence [1]. Local recurrences often result in repeated surgeries, which increase morbidity, and distant recurrence/metastasis are often fatal because of the ineffective chemotherapy [4]. Unfortunately, the molecular mechanisms underlying SACC progression and metastasis are still elusive, and further efforts are needed to uncover the molecular mechanisms, which may aid us with a better management of SACC.

MicroRNAs (miRNAs) belong to the family of non-coding RNAs and are ~ 22 nucleotides in length [5]. MiRNAs act as effective post-transcriptional mediators of gene expression via inducing targeted mRNAs degradation or translational repression [5]. Dysregulation of miRNAs has been identified in numerous human malignancies, and miRNAs play important roles in regulating human cancer progression and metastasis [6]. Unsurprisingly, the involvement of miRNAs in SACC progression and metastasis has also been reported. Liu et al. [7], identified the up-regulation of miR-155 in SACC tissues and found that miR-155 facilitated cell cycle progression and enhanced invasion of SACC. MiR-181a was found to be down-regulated in SACC cells with higher metastatic potential and inhibited SACC cell migration via targeting mitogen-activated protein kinase 1-Snai2 signaling axis [8]. Zhou et al. [9], also demonstrate that miR-122 inhibition was effective to induce cell apoptosis and attenuate cell migration of SACC cells. A recent study demonstrated that miR-93-5p promoted SACC cell proliferation, migration and invasion via targeting breast cancer metastasis suppressor 1 like [10]. In addition, a further study using miRNA array screening identified the down-regulation of miR-140-5p in SACC [11]. The tumor-suppressive role of miR-140-5p has been elucidated in various types of malignancies [12–16]. However, the involvement of miR-140-5p in SACC progression and metastasis has not been elucidated yet.

In the present study, we firstly examined the effects of miR-140-5p overexpression/knockdown on the proliferation, apoptosis and invasion of SACC cells. A further mechanistic investigation was carried out to determine the downstream targets of miR-140-5p. The role of miR-140-5p in in vivo tumor growth was also confirmed in a nude mice xenograft model. More importantly, the expression of miR-140-5p and its downstream mediators were further verified in the clinical sample tissues.

## Materials and methods

### Collection of clinical specimens

All the SACC clinical tissues and surrounding normal salivary gland tissues were collected from 35 patients who underwent surgical resection at Baoding No.1 Central Hospital between January 2016 and June 2019. The patients had no radiotherapy or chemotherapy before the surgeries. Ethical approval was obtained from the Ethics Committee of Baoding No.1 Central Hospital, and each patient signed the informed consent. All the collected samples were immediately frozen in liquid nitrogen and stored in − 80 °C before quantitative real-time PCR (qRT-PCR) analysis.

### Cell culture

The SACC cell lines (SACC-83 and SACC-LM) were obtained from the China Center for Type Culture Collection (Shanghai, China) and were grown in Dulbecco’s modified Eagle’s medium (DMEM; Sigma-Aldrich, USA) containing 10% fetal bovine serum (FBS; Thermo Fisher Scientific, USA). All the cells were kept under a humidified condition with 5% CO_2_ at 37 °C.

### Chemicals, miRNA mimics, inhibitors, plasmids and cell transfection

The survivin inhibitor, YM-155 were purchased from Sigma-Aldrich (St. Louis, USA), and the SACC-83 and SACC-LM were treated with 100 nM YM-155 for 24 h. The miRNA mimics and inhibitors for miR-140-5p as well as the respective negative controls (NCs) including mimics NC and inhibitors NC were purchased from Ribobio (Guangzhou, China). The plasmids (pcDNA3.1) overexpressing survivin were purchased from GenePharma (Shanghai, China) and pcDNA3.1 empty vector was used as the NC. The miRNAs and/or plasmids were transfected into SACC cells using Lipofectamine 2000 reagent (Invitrogen) by following the manufacturer’s protocol. At 24 h after transfection, SACC-83 and SACC-LM cells were harvested for further analysis.

### Isolation of RNA and qRT-PCR analysis

RNA isolation from cells and tissues was performed using the TRIzol reagent (Takara, Dalian, China). The reverse transcription of miRNAs into cDNA was performed using the MiR-X miRNA First Strand Synthesis kit (Clontech, USA). For the mRNA detection, the first strand cDNA synthesis kit (Takara) was used to transcribe mRNA into cDNA. The real-time PCR was performed on an ABI7900 PCR system (Applied Biosystems, Foster City, USA) using TB Green Premix EX Taq II kit (Takara). The U6 and β-actin were used as internal controls to normalize miR-140-5p and survivin expression levels, respectively. The 2^−ΔΔCt^ method was used to calculate the differential expression of miRNA and mRNA.

### Cell Counting Kit-8 (CCK-8) assay

Cell proliferative ability of SACC-83 and SACC-LM was evaluated using CCK-8 assay (Dojindo, Tokyo, Japan). Briefly, SACC-83 and SACC-LM cells after different treatments were harvested and were seeded on the 96-well plates. After a further incubation for 24, 48, 72 and 96 h, the cells were incubated with 10 µL CCK-8 solution for 2 h at 37 °C. After that, the cell proliferation was evaluated by measuring optical density at 450 nm using a microplate reader (Bio-Tek, Winooski, USA).

### Colony formation assay

Colony formation assay was used to assess SACC-83 and SACC-LM cell growth. Briefly, SACC-83 and SACC-LM cells after different treatments were seeded onto the 6-well plates and were cultured for 10 days with the culture medium being refreshed every 3 days. At the end of the experiments, the cultured cells were fixed with methanol and stained with 0.5% crystal violet for 20 min. The number of colonies with more than 50 cells was counted under a light microscope.

### Transwell invasion assay

Cell invasive abilities of SACC-83 and SACC-LM cells were determined using Transwell chambers (24-well insert; 8 μm pore size; Corning, USA) coated with Matrigel (Sigma-Aldrich). Briefly, the cells after different treatments were re-suspended in serum-free DMEM and were seeded onto the upper chamber coated with Matrigel, while the lower chamber was filled with DMEM supplemented with 10% FBS. After a further incubation for 24 h at 37 °C, the cells invaded into the lower side of the membrane was fixed with methanol for 10 min at room temperature and stained with 0.5% crystal violet for 20 min. The number of invaded cells were quantified under a light microscope.

### Flow cytometry

Apoptosis of SACC-83 and SACC-LM cells were detected using Annexin V-FITC/propidium iodide (PI) Apoptosis Kit (Thermo Fisher Scientific). After receiving different treatments, the SACC-83 and SACC-LM cells were harvested and washed with ice-cold phosphate buffered saline (PBS). After washing, the cells were incubated with binding buffer containing Annexin V and PI for 15 min at room temperature in a dark environment. The cell apoptosis was detected on a flow cytometer (BD Bioscience, USA) and was analyzed using the FlowJo™ software (BD Bioscience).

### Caspase-3 activity assay

The caspase-3 activity of SACC-83 and SACC-LM cells were measured using a Caspase-3 Activity Assay kit (Abcam, USA) according to the manufacturer’s protocol.

### Luciferase reporter assay

The luciferase reporter assay was used to determine whether miR-140-5p could directly target survivin. Wild type (WT) and mutant (MUT) survivin 3′ untranslated region (3′UTR) with miR-140-5p binding sites were amplified by PCR and the amplified fragments were inserted into the pSiCHeck-2 vector (Promega, USA) to generate the corresponding WT and MUT reporter vectors (Luc-survivin 3′UTR-WT and Luc-survivin 3′UTR-MUT). The dual luciferase reporter assay were performed in SACC-83 and SACC-LM cells after being co-transfected with corresponding miRNAs (mimics NC or miR-140-5p mimics) and reporter vectors (Luc-control, Luc-survivin 3′UTR-WT or Luc-survivin 3′UTR-MUT) using Lipofectamine 2000 reagent (Invitrogen). At 48 h after transfection, the Dual-Glo Luciferase Assay System (Promega) was utilized to measure the relative luciferase activity.

### Western blot analysis

Cells after different treatments were lysed using RIPA lysis buffer containing a protease inhibitor cocktail. The protein concentrations from the cell lysates were measured using a bicinchoninic acid protein assay (Bio-Rad, Hercules, USA). After electrophoresis on a 10% SDS-PAGE, the separated proteins were transferred to the PVDF membranes. After incubation with 5% skimmed milk at room temperature for 1 h, the PVDF membranes were incubated with corresponding primary antibodies including survivin, cleaved caspase-3/-9, X-linked inhibitor of apoptosis protein (XIAP), N-cadherin, vimentin, E-cadherin, matrix metallopeptidase (MMP)-2/-9 and β-actin at 4 °C overnight and then with horseradish peroxidase-conjugated secondary antibody for 2 h at room temperature. All the primary and secondary antibodies were purchased from Cell Signaling Technology (Danvers, USA). The immunoreactive bands were detected using the ECL kit (Thermo Fisher Scientific).

### Xenograft formation and in vivo tumor growth

The male BALB/c-nu/nu nude mice (4–6 weeks old) were purchased from the Vital River Laboratory (Beijing China), and all the animal experimental procedures were conducted under the approval of the Animal Ethics Committee of Baoding No.1 Central Hospital. For the miR-140-5p overexpression, the miR-140 sequence was amplified and inserted into the lentiviral expression vector PGMLV-CMV-MSC-EF1-ZsGreen1-Ta2A-Puro (Genomeditech, Shanghai, China). For miR-140 knockdown, the shRNA sequences were amplified and inserted into the pGML-SC5 RNAi lentiviral vector (Genomeditech). Lentivirus packaging and transfection were performed as previously described. Briefly, the SACC-LM cells with miR-140-5p overexpression/knockdown or the respective controls were re-suspended in 100 µL PBS and subcutaneously administered into right flank of the nude mice (each group had 5 animals). The tumor size was measured every 7 days for 5 weeks and was calculated using the following formula: tumor volume = width × width × length/2. At the end of the experiments, the tumors were harvested for further analysis.

### Statistical analysis

All the data were presented as mean ± standard deviation. Data analyses were conducted using GraphPad Prism Version 6.0 (GraphPad Software, La Jolla, USA). Student’s t test or one-way ANOVA followed by the Dunnett’s multiple comparison test was used to analyze the differences between/among different treatment groups. Correlation between two variables were analyzed by using Spearman Correlation analysis. P < 0.05 were considers as statistically significant.

## Results

### MiR-140-5p overexpression suppressed SACC-83 and SACC-LM cell proliferation, invasion and induced apoptosis, inhibited in vivo tumor growth

**Fig. 1:**
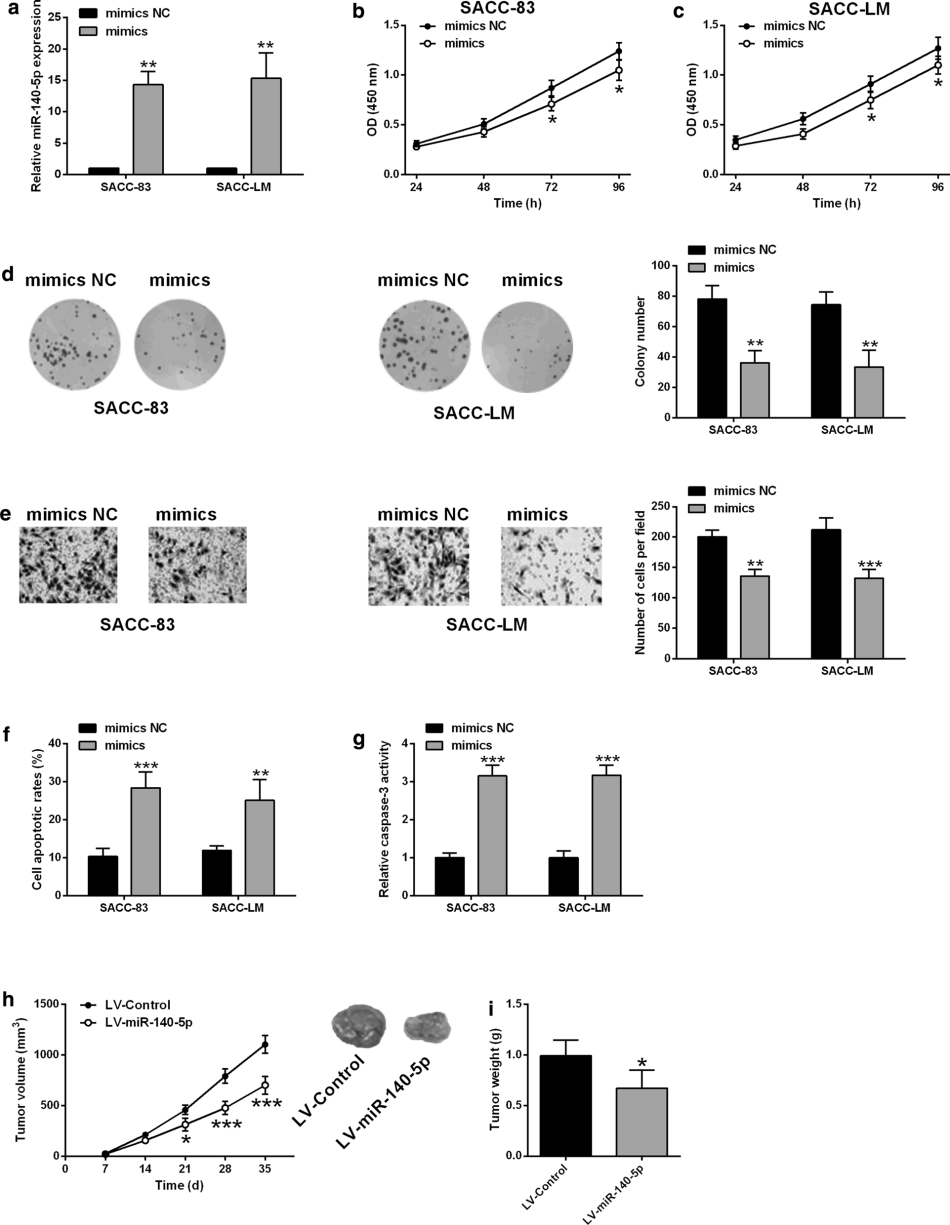
MiR-140-5p overexpression suppressed SACC-83 and SACC-LM cell proliferation, invasion and induced apoptosis, inhibited in vivo tumor growth. **a** qRT-PCR determined miR-140-5p expression levels in SACC-83 and SACC-LM cells after being transfected with mimics NC or miR-140-5p mimics (n = 3). **b**, **c** CCK-8 assay, **d** colony formation assay, **e** transwell invasion assay respectively determined cell proliferation, growth and invasion of SACC-83 and SACC-LM cells after being transfected with mimics NC or miR-140-5p mimics. **f** Flow cytometry and **g** caspase-3 activity assay respectively determine cell apoptotic rates and caspase-3 activity of SACC-83 and SACC-LM cells after being transfected with mimics NC or miR-140-5p mimics (n = 3). **h** In vivo tumor growth and (I) tumor weight from the xenograft nude mice inoculated with control SACC-LM cells or SACC-LM cells overexpressing miR-140-5p (n = 5). Significant difference between treatment groups were indicated as *P < 0.05, **P < 0.01 and ***P < 0.001

Firstly, we determined the effects of miR-1405-5p overexpression on the cell proliferation, invasion and apoptosis of SACC-83 and SACC-LM cells. The overexpression of miR-140-5p were detected in SACC-83 and SACC-LM cells with miR-140-5p mimics transfection when compared to mimics NC transfection (Fig. 1a). The CCK-8 assay showed that miR-140-5p overexpression suppressed the SACC-83 and SACC-LM cell proliferation (Fig. 1b, c). Further colony formation assay demonstrated that the number of colonies growing from SACC-83 and SACC-LM was significantly reduced by miR-140-5p overexpression when compared to mimics NC group (Fig. 1d). The transwell invasion assay determined the invasive ability of SACC-83 and SACC-LM cells after being transfected with mimics NC or miR-140-5p mimics, and miR-140-5p mimics transfection caused a significant decrease in the invasive cell number when compared to mimics NC group (Fig. 1e). The effects of miR-140-5p overexpression on SACC-83 and SACC-LM cell apoptosis were analyzed by flow cytometry and caspase-3 activity assay kit. As shown in Fig. 1f, g, miR-140-5p overexpression markedly increased cell apoptotic rates and caspase-3 activity of SACC-83 and SACC-LM cells (Fig. 1f, g). The effects of miR-140-5p overexpression were further evaluated in a nude mice inoculated with SACC-83 cells overexpressing miR-140-5p or control SACC083 cells, and miR-140-5p overexpression significantly attenuated the tumor growth of the nude mice (Fig. 1h), and the tumor weight was reduced in the LV-miR-140-5p group when compared to LV-control group (Fig. 1i).

### MiR-140-5p knockdown enhanced SACC-83 and SACC-LM cell proliferation, invasion and inhibited apoptosis, accelerated in vivo tumor growth

**Fig. 2:**
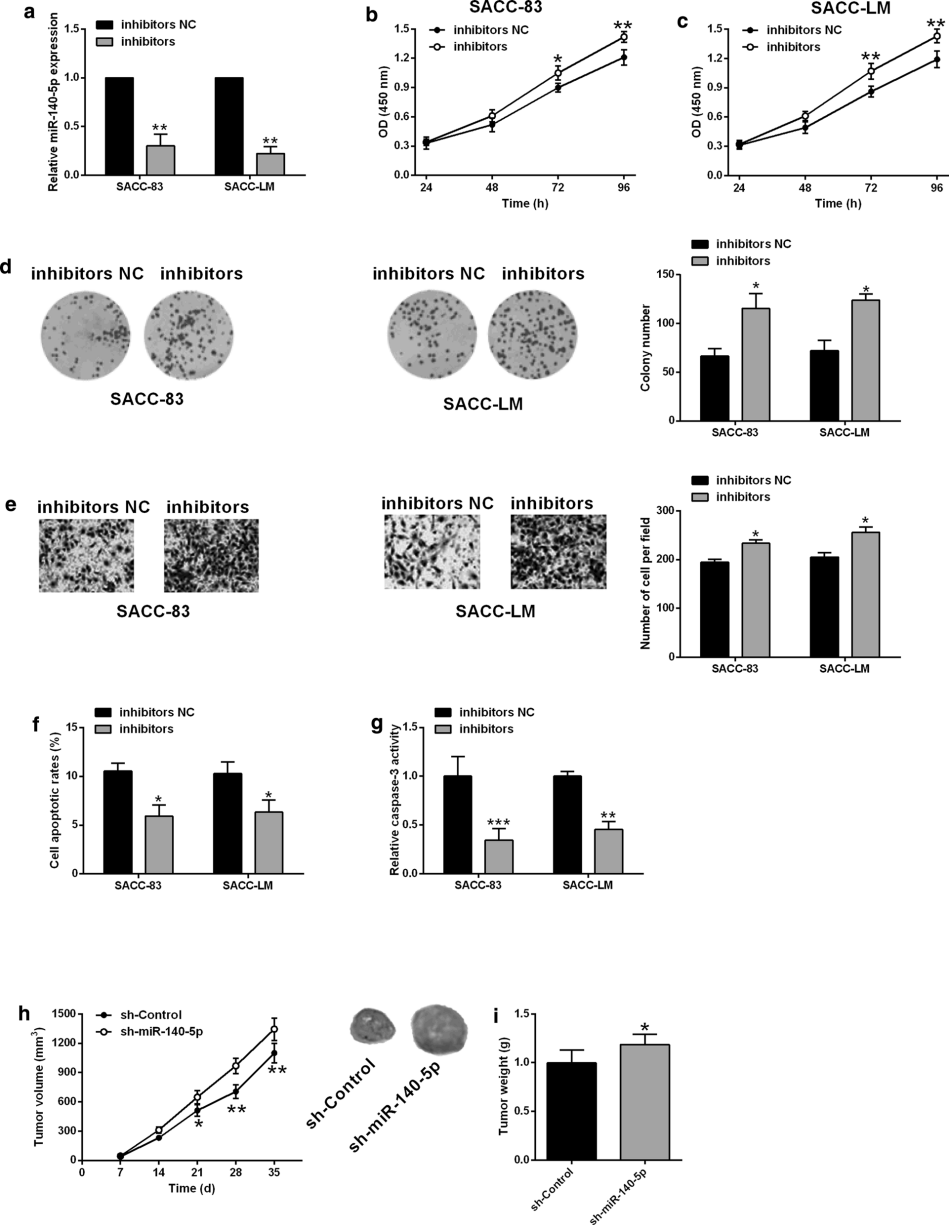
MiR-140-5p knockdown enhanced SACC-83 and SACC-LM cell proliferation, invasion and inhibited apoptosis, accelerated in vivo tumor growth. **a** qRT-PCR determined miR-140-5p expression levels in SACC-83 and SACC-LM cells after being transfected with inhibitors NC or miR-140-5p inhibitors (n = 3). **b**, **c** CCK-8 assay, **d** colony formation assay, **e** transwell invasion assay respectively determined cell proliferation, growth and invasion of SACC-83 and SACC-LM cells after being transfected with inhibitors NC or miR-140-5p inhibitors. **f** Flow cytometry and **g** caspase-3 activity assay respectively determine cell apoptotic rates and caspase-3 activity of SACC-83 and SACC-LM cells after being transfected with inhibitors NC or miR-140-5p inhibitors (n = 3). **h** In vivo tumor growth and **i** tumor weight from the xenograft nude mice inoculated with control SACC-LM cells or SACC-LM cells with miR-140-5p silencing (n = 5). Significant difference between treatment groups were indicated as *P < 0.05, **P < 0.01 and ***P < 0.001

The effects of miR-140-5p knockdown on SACC cancer progression were evaluated using both in vitro and in vivo functional assays. As show in Fig. 2a, miR-140-5p inhibitors transfection significantly reduced miR-140-5p expression levels when compared to inhibitors NC transfection (Fig. 2a). The CCK-8, colony formation and transwell invasion assays showed that miR-140-5p knockdown potentiated cell proliferation, growth and invasion of SACC-83 and SACC-LM cells (Fig. 2b–e). The flow cytometry and caspase-3 activity assay showed that miR-140-5p knockdown inhibited cell apoptosis and caspase-3 activity of SACC-83 and SACC-LM cells (Fig. 2f, g). Further in vivo experiments showed that miR-140-5p knockdown enhanced the in vivo growth of SAC-83 cells (Fig. 2h, i).

### MiR-140-5p targeted survivin 3′UTR and suppressed survivin expression

**Fig. 3:**
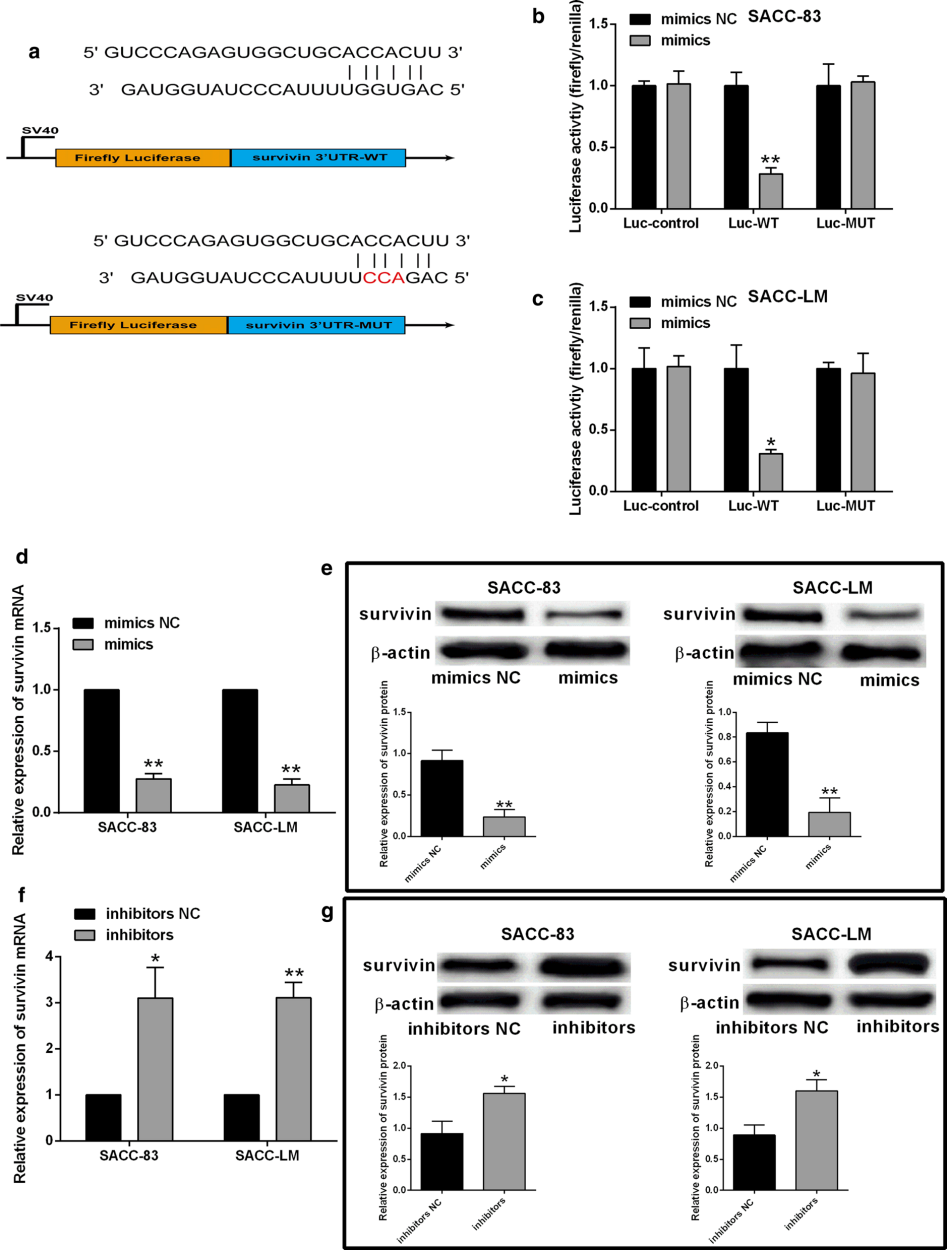
MiR-140-5p targeted survivin 3′UTR and suppressed survivin expression. **a** Putative binding sites between miR-140-5p and survivin 3′UTR as predicted using online StarBase 2.0 tool. **b**, **c** Relative luciferase activity of different reporter constructs in SACC-83 and SACC-LM cells after being transfected with mimics NC or miR-140-5p mimics. **d**, **e** qRT-PCR and western blot assays respectively determined survivin mRNA and protein expression levels in SACC-83 and SACC-LM cells after being transfected with mimics NC or miR-140-5p mimics. **f**, **g** qRT-PCR and western blot assays respectively determined survivin mRNA and protein expression levels in SACC-83 and SACC-LM cells after being transfected with inhibitors NC or miR-140-5p inhibitors. N = 3. Significant difference between treatment groups were indicated as *P < 0.05 and **P < 0.01

The potential binding sites between miR-140-5p and survivin 3′UTR was predicted using Starbase V2.0 online database (http://starbase.sysu.edu.cn/). The predicted sites were shown in Fig. 3a, and the predicted binding sites were subjected to site-directed mutagenesis to generate the fragments of the mutated survivin 3′UTR (Fig. 3a). The luciferase reporter assay showed that miR-140-5p overexpression suppressed the luciferase activity of Luc-survivin 3′UTR-WT when compared to mimics NC group (Fig. 3b, c); while the luciferase activity of Luc-control and Luc-survivin 3′UTR –MUT was unaffected by miR-140-5p overexpression (Fig. 3b, c). The qRT-PCR and western blot assays determined the effects of miR-140-5p overexpression/knockdown on survivin expression. As shown in Fig. 3d, e, miR-140-5p overexpression suppressed the survivin mRNA and protein expression levels in SACC-83 and SACC-LM cells (Fig. 3d, e); on the other hand, miR-140-5p knockdown up-regulated survivin expression in SACC cells (Fig. 3f, g).

### MiR-140-5p regulated SACC-83 and SACC-LM cancer cell progression via targeting survivin

**Fig. 4:**
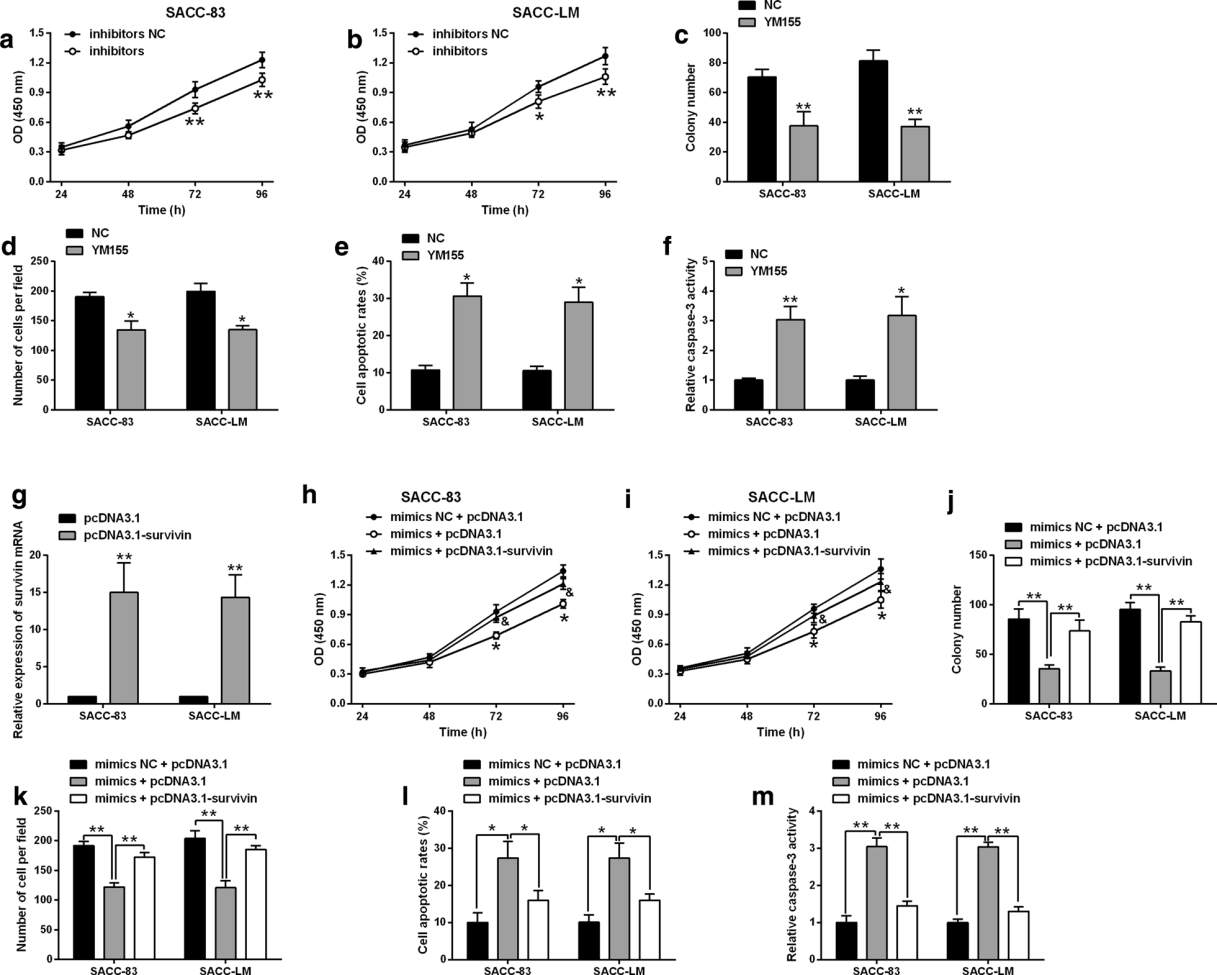
MiR-140-5p regulated SACC-83 and SACC-LM cancer cell progression via targeting survivin. **a**, **b** CCK-8 assay, **c** colony formation assay, **d** transwell invasion assay respectively determined cell proliferation, growth and invasion of SACC-83 and SACC-LM cells after being treated with 100 nM YM-155 or normal medium (NC). **e** Flow cytometry and **f** caspase-3 activity assay respectively determine cell apoptotic rates and caspase-3 activity of SACC-83 and SACC-LM cells after being treated with 100 nM YM-155 or normal medium (NC). **g** qRT-PCR determined survivin mRNA expression levels in SACC-83 and SACC-LM cells after being transfected with pcDNA3.1 or pcDNA3.1-survivin. **h**, **i** CCK-8 assay, **j** colony formation assay, **k** transwell invasion assay respectively determined cell proliferation, growth and invasion of SACC-83 and SACC-LM cells after being co-transfected with mimics NC + pcDNA3.1, miR-140-5p mimics + pcDNA3.1 or miR-140-5p mimics + pcDNA3.1-survivin. **f** Flow cytometry and **g** caspase-3 activity assay respectively determine cell apoptotic rates and caspase-3 activity of SACC-83 and SACC-LM cells after being co-transfected with mimics NC + pcDNA3.1, miR-140-5p mimics + pcDNA3.1 or miR-140-5p mimics + pcDNA3.1-survivin. N = 3. Significant difference between treatment groups were indicated as *P < 0.05 and **P < 0.01

The effects of survivin inhibition on SACC-83 and SACC-LM proliferation, invasion and apoptosis were determined by in vitro functional assays. YM-155, the inhibitor of survivin, significantly suppressed SACC-83 and SACC-LM cell proliferation, growth and invasion (Fig. 4a–d). In addition, YM-155 treatment increased cell apoptotic rates and caspase-3 activity of SACC-83 and SACC-LM cells (Fig. 4e, f).

As survivin inhibition repressed SACC-83 and SACC-LM cell progression, we further determined if enforced expression of survivin could counteract the tumor-suppressive effects of miR-140-5p overexpression in SACC-83 and SACC-LM cells. The enforced expression of survivin significantly attenuated the inhibitory effects of miR-140-5p overexpression on SACC-83 and SACC-LM cellproliferation, growth and invasion (Fig. 4g–j), and counteracted the enhanced effects of miR-140-5p overexpression on SACC-83 and SACC-LM cell apoptosis (Fig. 4k–m).

**Fig. 5:**
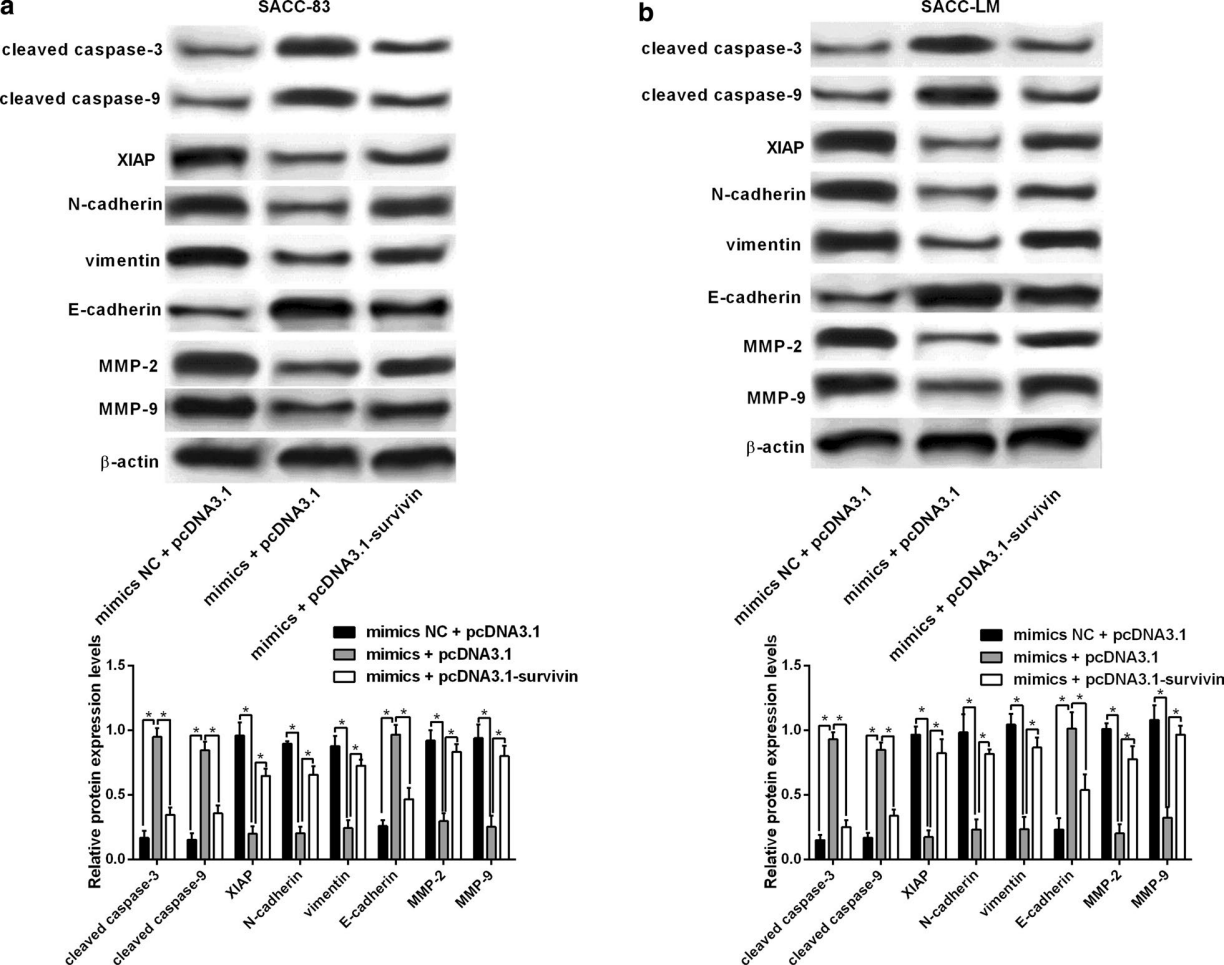
MiR-140-5p modulated the protein expression levels of apoptosis- and epithelial-mesenchymal transition-related mediators as well as matrix metallopeptidase-2/-9 via targeting survivin. Western blot assay determined protein expression levels of cleaved caspase-3/-9, XIAP, N-cadherin, vimentin, E-cadherin, matrix metallopeptidase-2/-9 in SACC-83 and SACC-LM cells after being co-transfected with mimics NC + pcDNA3.1, miR-140-5p mimics + pcDNA3.1 or miR-140-5p mimics + pcDNA3.1-survivin

Furthermore, the western blot assay were performed to determine the protein expression levels of mediators related to cell apoptosis and cell invasion. MiR-140-5p overexpression significantly increased the protein levels of cleaved caspase-3 and -9, but decreased the XIAP protein levels, which was attenuated by the enforced expression of survivin in SACC-83 and SACC-LM cells (Fig. 5a, b). MiR-140-5p overexpression down-regulated N-cadherin and vimentin, but up-regulated E-cadherin, and this effect was partially reversed by survivin overexpression (Fig. 5a, b). As MMPs are important in regulating cancer cell invasion and migration, we further examined the protein levels of MMP-2 and MMP-9 in SACC-83 and SACC-LM cells. MiR-140-5p overexpression reduced MMP2 and MMP9 protein levels, which were counteracted by the enforced expression of survivin in SACC-83 and SACC-LM cells (Fig. 5).

### Expression of miR-140-5p and survivin mRNA in SACC clinical samples

**Fig. 6:**
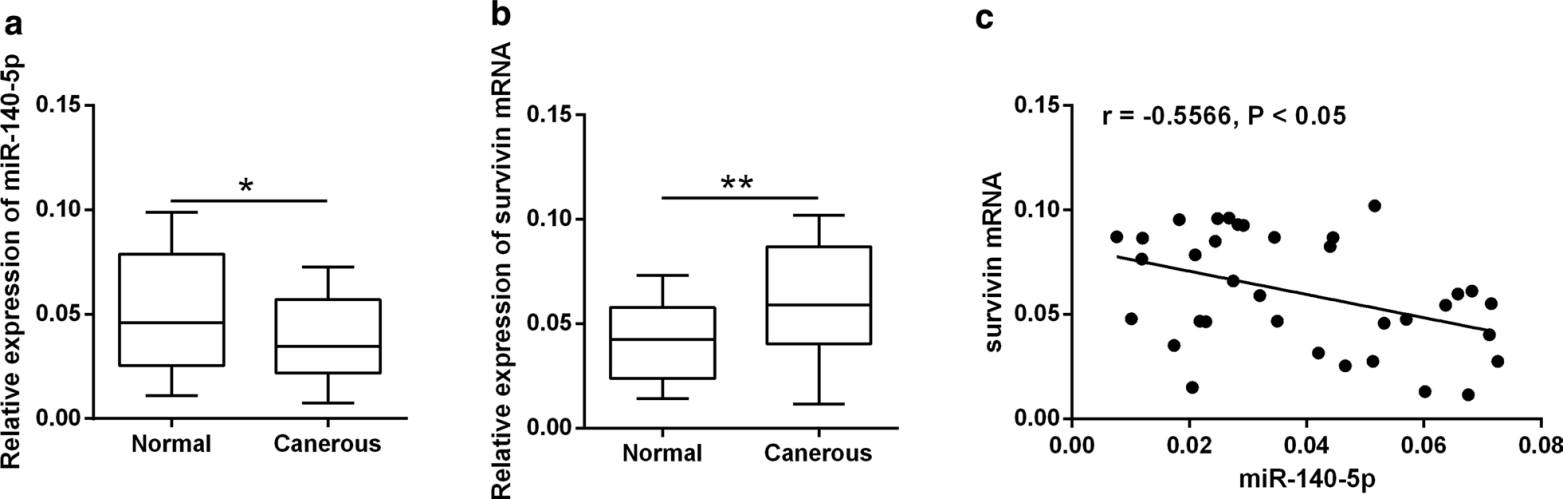
Expression of miR-140-5p and survivin mRNA in SACC clinical samples. **a**, **b** qRT-PCR determined the miR-140-5p and survivin mRNA expression levels in SACC tissues and normal surround tissues from 35 patients with SACC. **c** Correlation analysis between miR-140-5p expression levels and survivin mRNA expression in SACC tissues. *P < 0.05 and **P < 0.01

The expression of miR-140-5p and survivin mRNA in SACC tissues and surrounding normal salivary tissues from 35 SACC patients were subjected to the qRT-PCR analysis. MiR-140-5p was down-regulated and survivin mRNA was up-regulated in the SACC tissues when compared to normal tissues (Fig. 6a, b), and miR-140-5p expression level was inversely correlated with survivin mRNA expression level in SACC tissues (Fig. 6c).

## Discussion

The patients with malignant SACC usually had distal metastasis, which led to poor prognosis of these patients [2]. Due to the limited understanding of the molecular mechanisms underlying SACC progression and metastasis, the current treatment regimens for SACC remain unsatisfactory [2]. Therefore, it is urgent for us to develop novel targets for the better management of this malignancy. In the present study, we showed that miR-140-5p overexpression suppressed SACCC cell proliferation and invasion, induced cell apoptosis and inhibited in vivo tumor growth of SACC cells. The loss-of-function studies showed that miR-140-5p knockdown induced enhanced SACC cell proliferation and invasion, inhibited cell apoptosis and led to accelerated in vivo tumor growth. The bioinformatics prediction and luciferase reporter assay revealed that miR-140-5p directly targeted survivin 3′UTR, and survivin was inversely regulated by miR-140-5p. Knockdown of survivin exerted tumor-suppressive effects on SACC cells, while enforced expression of survivin counteracted the tumor-suppressive actions of miR-140-5p overexpression in SACC cells. More importantly, the down-regulation of miR-140-5p and the up-regulation of survivin were detected in the SACC clinical tissues, and miR-140-5 expression was inversely correlated with survivin mRNA expression level in SACC tissues. Our study for the first time elucidated the role of miR-140-5p in SACC progression.

Up to date, accumulating evidence reported the tumors-suppressive role of miR-140-5p in different types of human malignancies. MiR-140-5p was down-regulated in hepatocellular carcinoma (HCC) tissues and was correlated with poor prognosis of HCC patients [17]. In addition, miR-140-5p suppressed HCC growth and metastasis via regulating transforming growth factor β receptor 1 and fibroblast growth factor 9 [17]. MiR-140-5p was down-regulated in hypopharyngeal squamous cell carcinoma (HSCC) tissues and cell lines and restoration of miR-140-5p in HSCC cells repressed tumor cell invasion and migration via targeting ADAM10/Notch1 axis [18]. Fang et al., showed that down-regulated miR-140-5p expression was detected in the cancerous gastric clinical samples and was correlated with shorter overall survival of gastric cancer patients; mechanistically, miR-140-5p exerted its tumor-suppressive effects on gastric cancer via targeting YES1 and THY/Notch signaling [16, 19]. A recent study by Liu et al. [13], showed that miR-140-5p was down-regulated in Wilms’ tumor and suppressed tumor progression via modulating TGFBRI/SMAD2/3 and IGF-1R/AKT signaling pathways. The miRNA array analysis of SACC clinical samples showed the down-regulation of miR-140-5p in the malignant SACC tissues [11]. In our study, we consistently found that miR-140-5p acted as a tumor suppressor in SACC via inhibiting SACC cell proliferation and invasion, increasing cell apoptosis and attenuating the in vivo tumor growth of SACC-LM cells.

Survivin belongs to the family member of the apoptosis inhibitor and is encoded by the baculoviral inhibitor of apoptosis repeat-containing 5 [20]. Survivin has been reported to play important roles in regulating cell survival, apoptosis and cell cycle by acting with the apoptosis effectors in various types of malignancies [20]. Recent studies found that survivin was highly expressed in SACC tissues and was correlated with poor prognosis in the patients with SACC [21–23]. In our study, bioinformatics prediction and luciferase reporter assay showed that survivin was targeted and inversely regulated by miR-140-5p in SACC cells. Further in vitro functional assays found that inhibition of survivin suppressed SACC cell proliferation and invasion, and also induced cell apoptosis. On the other hand, we found that the enforced expression of survivin counteracted the tumor suppressive effects of miR-140-5p in SACC cells. In fact, the oncogenic role of survivin has been implicated in the SACC cells in several studies. Simvastatin suppressed SACC cell proliferation and invasion via repressing the survivin expression [24]. Selective inhibition of survivin by YM155 and siRNA significantly inhibited SACC cell proliferation and induced autophagy-dependent cell death [25, 26]. Collectively, these data may imply that miR-140-5p suppressed SACC cell proliferation and invasion via modulating survivin expression. Furthermore, we determined the downstream apoptosis effectors of survivin [27], and our results showed that miR-140-5p increased protein levels of cleaved caspase-3/-9, and decreased XIAP protein levels, which were attenuated by enforced expression of survivin in SACC cells. As deregulation of EMT and MMPs have been shown to contribute to metastasis of malignancies including SACC [28–30]. In our study, we determined the EMT-related mediators including E-cadherin, N-cadherin and vimentin as well as MMP-2/-9, and our data showed that miR-140-5p overexpression inhibited EMT and repressed protein levels of MMP2 and MMP9, which was counteracted by survivin overexpression in SACC cells. In fact, miR-140-5p was effective to inhibit EMT in esophagus cancer [31] and miR-140-5p also inhibited glioma metastasis via repressing MMP2 expression [32]. Taken together, the tumor suppressive effects of miR-140-5p on SACC may be related to regulation of apoptosis- and EMT-related mediators as well as MMPs.

## Conclusions

In conclusion, the present study demonstrated the tumor-suppressive effects of miR-140-5p in SACC. MiR-140-5p suppressed SACC cell proliferation and invasion, induced cell apoptosis via regulating survivin expression. The present study provided evidence that that miR-140-5p could be promising target for treating SACC, which requires further investigations.

## Data Availability

All the data in the manuscript are available upon reasonable request.

## Abbreviations

3′UTR: 3′ untranslated region
CCK-8: cell counting kit-8
DMEM: Dulbecco’s modified Eagle’s medium
EMT: epithelial-mesenchymal transition
FBS: fetal bovine serum
miRNA: microRNA
HSCC: hypopharyngeal squamous cell carcinoma
MMP: matrix metallopeptidase
MUT: mutant
NC: negative control
PI: propidium iodide
qRT-PCR: quantitative real-time PCR
SACC: salivary adenoid cystic carcinoma
WT: wild type
XIAP: X-linked inhibitor of apoptosis protein
